# Effectiveness of the mobile application Holidaily in reducing work-related rumination when returning to work after vacation: a randomized controlled trial

**DOI:** 10.3389/fdgth.2026.1698339

**Published:** 2026-04-22

**Authors:** Alexandra Thomsen, Christine Syrek, Hanna A. Brückner, Jessica de Bloom, Monique Janneck, Markus Domin, Jo Annika Reins, Dirk Lehr

**Affiliations:** 1 Department of Health Psychology and Applied Biological Psychology, Institute of Sustainability Psychology, Leuphana University, Lüneburg, Germany; 2 Department of Management Sciences, University of Applied Sciences Bonn-Rhein Sieg, Rheinbach, Germany; 3 Department of Human Resource Management and Organisational Behavior, University of Groningen, Groningen, Netherlands; 4 Department of Psychology, Tampere University, Tampere, Finland; 5 Institute for Interactive Systems, Technische Hochschule Luebeck, Luebeck, Germany

**Keywords:** digital mental health, work-related rumination, mobile intervention, recovery, recreational activities, vacation, workers, mental detachment

## Abstract

**Background:**

Vacations reliably improve indicators of mental health, largely by providing relief from work-related stress. Low levels of work-related rumination, a key transdiagnostic factor linked to burnout and depression, are considered prerequisites for successful recovery both during vacations and in daily working life. However, such benefits are typically short-lived, with a rapid “fade-out” upon return to work. To address this challenge, we developed Holidaily, a low-threshold, gamified mobile health intervention designed to translate recovery science into daily digital practice and sustain the mental health gains of vacations.

**Methods:**

In a randomized controlled trial (RCT), Holidaily was evaluated as a digital mental health intervention targeting work-related rumination, the primary outcome. Assessments were conducted two weeks prior to vacation and two weeks after the return to work, before waitlist controls were granted access. Given the novelty of the research, a wide range of exploratory outcomes was also assessed.

**Results:**

A total of 190 workers from the general population were randomized to either the intervention (*n* = 91) or waitlist control group (*n* = 99). ANCOVA, in accordance with the intention-to-treat principle, indicated that the intervention group reported significantly lower levels of work-related rumination at two weeks post-vacation compared with controls [*p* < 0.001, *d* = −0.67 (−1.0; −0.4)]. At this time, rumination levels were still reduced by 22.2% in the intervention group, compared with 6.9% in controls relative to baseline. Among app users, reductions persisted for up to four weeks (26.1%). Sensitivity analyses confirmed these results. These findings provide first evidence that a mobile health technology can extend vacation-related recovery benefits and reduce work-related rumination in workers.

**Conclusions:**

This is the first RCT to show that the rapid fade-out of vacation benefits is not inevitable. Holidaily appears to improve workers’ ability to reduce levels of work-related rumination. These results highlight the potential of scalable digital interventions to foster sustainable mental health in working populations and support preventive public health efforts.

**Clinical Trial Registration:**

https://drks.de/search/de/trial/DRKS00013650, German WHO DRKS00013650.

## 1 Background

The beneficial influence of recovering sufficiently from occupational stress is manifold and has recently received considerable attention in both practice and research, as reflected in several meta-analyses (1–3). Recovery, described as the process of unwinding and restoring from strain caused by work-related stressors (4), usually takes place during leisure time, that is, after work, on weekends, and especially during vacations (5). In particular, vacations, which typically offer a prolonged break from work, have emerged as promising times for recovery and have been shown to enhance well-being and life satisfaction (6) and reduce symptoms of emotional exhaustion and depression (5, 7–10).

Despite these positive effects, it remains unclear how long the benefits of vacations persist after returning to work. The first meta-analysis on this issue, conducted by de Bloom et al. (11), revealed that while vacation improved life satisfaction and physical health, these effects tended to diminish within two to four weeks after returning to work. Recent findings support this trend. In a meta-analysis of 13 observational studies investigating the impact of vacations on workers' well-being and the fading of this effect, the results suggest that the benefits to well-being were no longer present in the second week post-vacation, with scores continuously decreasing (12). However, these findings were challenged by Grant, Buchanan, and Shockley (13), who conducted a larger meta-analysis that included observational (*k* = 31) and intervention (*k* = 1) studies. Their results suggest that although the vacation effect declines, the rate of decline is slower than previously assumed, with well-being levels remaining elevated compared to pre-vacation.

Although these meta-analyses provide valuable insights, their findings must be interpreted with caution. Notably, only one randomized controlled trial (RCT) was included across these reviews: Blank et al. (14) investigated the effects of a four-night stay in a hotel compared with a four-night vacation at home (“staycation”) in 40 middle managers. While no differences between the modes of vacation were observed, positive effects on recovery, perceived stress, strain, and well-being were found for both groups, which lasted for several weeks after the vacation ended. Although this was one of the first RCTs to explore the fading of the vacation effect, these results are limited in generalizability because of the small, homogenous sample and controlled experimental conditions. Considering that only one RCT was included across all three meta-analyses, there is a pressing need for more rigorous controlled investigations into the effects of vacations, as emphasized by Speth, Wendsche, and Wegge (12). A further limitation of these meta-analyses is the large heterogeneity of measurement intervals in the primary studies, making it very difficult to compare outcomes and identify specific effects, such as the vacation effect and the fading of this effect. In response to this issue, Grant, Buchanan, and Shockley (13) recommended more standardized measurement protocols. This recommendation is reflected in two observational studies on the fade-out of vacation benefits (15, 16), which used weekly assessments, such as, two weeks before, during and two weeks after vacation. Their findings suggest that post-vacation benefits may diminish during the first weeks after returning to work. In particular, Flaxman et al. (16) found that some outcomes approached baseline levels within two weeks of work resumption. Against this background, assessing outcomes two weeks post-vacation appears to be a particularly informative and conservative way to examine whether beneficial effects persist beyond the immediate post-vacation period.

Furthermore, the magnitude and speed at which the beneficial effects fade seem to depend on several factors, including (1) pre-vacation workplace characteristics, (2) methodological study design, that is, the timing of baseline assessment, and (3) post-vacation recovery behavior. Specifically, (1) pre-vacation stressors, such as work overload, time pressures, work demands, and interpersonal conflicts, can lead to elevated stress symptoms, which have been found to accelerate the decrease in beneficial effects (17). (2) Methodological inconsistencies, particularly in the timing of baseline assessment, may also lead to confounded interpretations. For example, measuring well-being immediately before a vacation may capture the acute stress of trip preparation rather than participants' typical levels of well-being during regular workdays, potentially inflating post-vacation effects (18). To mitigate this, it is recommended that baseline levels be assessed approximately two weeks prior to the vacation (9, 11). (3) In terms of post-vacation recovery behavior, two prospective studies indicated that maintaining recovery-related behavior post-vacation can help sustain the recovery experience (19, 20). Kühnel and Sonnentag (7) reported that among 131 teachers, those who maintained recovery behaviors beyond their vacation and during their daily working life prolonged the benefits of vacation compared with those who ceased such involvement. However, this line of evidence is primarily observational, and to date, Blank et al. (14) are the only researchers to have conducted an RCT investigating the impact of post-vacation recovery.

Typically, recovery involves engaging in recreational activities (e.g., spending time with friends/family, reading a book, exercising), and according to the Stressor-Detachment-Model (21) and the DRAMMA-Model (22), such activities can be attributed to one of several recovery experiences, for instance, (mental) detachment, autonomy, mastery, meaning, and affiliation. Among these, mental detachment from work-related stressors is considered the most important experience and has received the most research attention to date, as indicated by meta-analyses e.g., (1–3). Particularly during vacations, workers appear to mentally detach more effortlessly, which could be explained by the physical distance to their daily working life. Through the process of mentally detaching from work, the restoration of depleted resources during effort expenditure can occur (23).

Notably, (lack of) detachment as a construct has no valence [“I forget about work”, (24)] and could theoretically arise from both repetitive negative thinking about work-related problems (25) and constructive problem solving, even from positive thinking about work (26). Within this broader approach to work-related thoughts, work-related rumination can be considered a domain-specific manifestation of repetitive negative thinking (27). This term was introduced by Harvey (28) and Ehring and Watkins (29) to describe a transdiagnostic risk factor for mental health conditions. Empirically, meta-analyses have demonstrated that lower levels of detachment are associated with higher levels of exhaustion and depression (1, 3, 30). Jimenez, Hu and Xu (31) reported a strong negative correlation between detachment and negative work-related thinking, which might be explained by the greater salience of the negative (32), leading to intuitive answers to detachment questions in light of problems at work. Although a lack of detachment is consistently associated with poor mental health, negative work-related thinking, particularly work-related rumination, is even more strongly associated with adverse mental health outcomes (31).

Work-related rumination, conceptualized as cognitive irritation (25), affective rumination (26), or depressive rumination (33), shares the characteristic that cognitions are negative, focused on problems, repetitive, partly intrusive, and difficult to disengage from (29). From a stress research perspective, Brosschot, Gerin and Thayer (34) emphasized the negative and repetitive nature of rumination as negatively toned, repetitive thought processes, theorizing that these processes result in “repeated or chronic activation of the cognitive representation of one or more psychological stressors”, which in turn are assumed to result in mental or physical illnesses. One meta-analysis confirmed substantial associations between work-related rumination and both negative affectivity and burnout (31). In general, repetitive negative thinking, including work-related rumination, has been shown to aggravate and maintain symptoms of depression, insomnia severity, and anxiety, and high levels of work-related rumination also seem to interfere with individuals' ability to recover from work stressors (26).

One meta-analysis (2) investigated the effectiveness of interventions targeting work-related thinking. Their findings suggest that an improvement in mental detachment and a reduction in rumination can be attained by increasing workers' sleep quality, stress management skills, mindfulness practices, and boundary management, and by decreasing workplace embitterment. In a subgroup analysis for negative work-related thinking, that is, rumination, a moderate effect was also revealed (*d* = 0.35). More specifically, of the 30 RCT studies included in the meta-analysis, only four incorporated the elements of smartphone-based interventions. However, none of these mobile applications (apps) specifically targeted work-related rumination. Instead, work-related rumination has only been examined in web-based interventions focused on occupational stress (35–37). Their findings suggest efficacy, with effects ranging between *d* = 0.35 and 0.54 for rumination assessed with the Irritation Scale (25). Further web-based RCTs targeting recovery from occupational stress reported greater effects, ranging from *d* = 0.73 and 1.06 for rumination (23, 38, 39). Notably, in the context of digital mental health interventions, web-based interventions are usually high-intensity, often involve guided elements such as feedback from digital coaches, and require substantial participant engagement compared with low-intensity interventions, such as apps. Nonetheless, meta-analytic findings indicate that even these lower-intensity mobile interventions can yield significant improvements in perceived stress among both clinical and non-clinical working populations (40, 41). Building on the potential of mobile interventions, a first attempt has been made to explore whether an app can prolong the benefits of vacations. Two studies were conducted using a beta version of the smartphone-based Holidaily 1.0 intervention, which was designed to encourage users to continue engaging in recovery activities both before and after vacations. While Smyth et al. (20) were open to the general working population (*N* = 171), Virtanen et al. (19) included a sample of only teachers (*N* = 79). The results were consistent, demonstrating that the advantageous impact of vacations was significantly greater than that at baseline for Smyth et al. (20), and positive for Virtanen et al. (19).

Importantly, these findings are especially noteworthy as the studies followed de Bloom et al.'s (11) recommendation to assess the baseline two weeks before vacation, thereby minimizing confounding due to pre-vacation stress. However, although these studies provide new insights concerning the course of recovery and indicate the potential of apps, the lack of a control condition is limiting, as it is unclear whether the beneficial effect can be attributed to the intervention itself or to the natural recovery effects of vacationing. Therefore, a pragmatic RCT design is needed to rigorously test whether smartphone-based interventions can effectively sustain and prolong the beneficial effects of vacations.

### 1.1 Study aim

This study aimed to conduct the first RCT to examine the efficacy of a gamified smartphone-based intervention in sustaining and enhancing the beneficial effects of vacations on workers' recovery and mental health. Specifically, the intervention targeted work-related rumination, a key transdiagnostic risk factor for mental health conditions. The intervention involved promoting engagement in recovery behaviors via the Holidaily-app 2.0, starting two weeks prior to the vacation and continuing post-vacation. Furthermore, this study also addresses several methodological limitations identified in previous research. Specifically, outcome measures were collected at multiple time points, including two weeks before vacation (to minimize potential confounding due to pre-vacation stress), the last working day, mid-vacation, the first day after work, and two weeks after vacation, to capture the trajectory of change across the vacation period. Additionally, the intervention group completed a sixth measurement four weeks post-vacation to explore longer-term effects; however, this time point was not assessed in the control group whereby limiting any causal interpretation of longer-term effects. Primarily the four-week follow-up was chosen to gather preliminary longer-term data that could inform hypothesis generation and the design of future studies. Consequently, the two-week post-vacation measurement serves as the primary outcome, aligning with prior evidence suggesting that vacation benefits typically diminish within the first two weeks of returning to work. Moreover, the study combined a rigorous experimental design with a low-intensity, scalable digital intervention to provide evidence on whether a mobile approach can prolong the beneficial effects of vacations and reduce work-related rumination, thereby contributing to workers’ sustainable recovery and mental well-being.

## 2 Method

### 2.1 Study design

This two-armed randomized controlled trial evaluated the efficacy of the mobile intervention “Holidaily 2.0” relative to a waitlist control group. The study protocol provided a detailed description of the design of the study (20). Analyses will be reported according to the “Consolidated Standards of Reporting Trials” (CONSORT) (42). Outcome measures were assessed online two weeks prior to vacation, on the last working day, in the middle of vacation, at the end of the first day working post-vacation, and two weeks after the vacation ended. The intervention group completed an additional follow-up assessment four weeks post-vacation (extended follow-up). At two weeks post-vacation, after completing the final questionnaire, the control group received access to the Holidaily-app through an automated code sent via email.

The two-week post-vacation assessment was pre-specified as the primary timepoint in the trial registration. It was selected based on evidence that vacation-related improvements diminish rapidly after returning to work, often approaching baseline within the first weeks (11, 43). We assumed that, within two weeks of returning to work, the short-lived benefits of vacation would have largely attenuated, thereby providing a suitable window in which any incremental effects of the intervention, particularly its potential to sustain reductions in work-related rumination beyond natural post-vacation recovery, would be detectable. Although a longer follow-up period would have provided additional information, it was not prioritized in this initial trial. The main reasons were to avoid placing an additional burden on the participants and to reduce the risk of dropout for those in the control group who would have otherwise waited longer to access the intervention.

### 2.2 Changes to the study protocol

Several deviations from the study protocol must be noted. First, the latest version of the CONSORT was used (42), replacing the older CONSORT-EHEALTH guideline (44) mentioned in the protocol. Second, recruitment was stopped earlier than anticipated. Despite the vast media interest, participant recruitment was more difficult and slower than expected. Recruitment difficulties were exacerbated by the COVID-19 pandemic and related travel restrictions, which led to the decision to end recruitment in spring 2021. Third, while the study was initially powered to assess differences between groups, the final sample size was insufficient to reliably calculate further analyses, such as moderation effects, which were specified in the study's protocol. Nevertheless, the study provides valuable insights into the feasibility and potential effectiveness of smartphone-based interventions in extending vacation benefits in real-world conditions.

### 2.3 Procedure

Recruitment and study execution took place between the summer of 2017 and spring 2021. While recruitment remained open during the beginning of the COVID-19 pandemic, participants did not register during this time, we therefore did not control for potential cohort effects. Participants from the general working population were recruited through on-air media and related websites. The most read health-related magazine in Germany (Apotheken Umschau), with 18.71 million readers, published an article on dealing with the fading of the beneficial vacation effect, which included a link to participate in the study. Anonymous registration took place either via https://www.holidaily.de or by providing the research team with their e-mail address directly. Potential participants received study details on the background, objectives, and procedures of the study via email. Prior to participation, an online screening questionnaire assessing the level of work-related rumination and vacation period and a signed consent form were submitted. Participants were required to submit a signed consent form to ensure informed consent. All data were collected and stored in accordance with the applicable data protection regulations, ensuring participant anonymity and confidentiality. The study was approved by the Ethics Committee of Leuphana University in Lueneburg (reference number: 201606, EB-Antrag Lehr201606_holidaily) and was conducted in compliance with ethical standards for research involving human participants.

The intervention group received access to the Holidaily-app two weeks prior to their vacation. After logging in, users created a customized profile with the following information: vacation location, first and last days of work, and first and last days of vacation. Once these steps were completed, users could start using the app by selecting Dailys and keeping track of their well-being.

Participants in the control group were invited to answer identical questionnaires following the same timeframe as the intervention group and received access to the app two weeks after returning to work after the vacation. In addition, the waitlist controls received personalized, automated feedback on their reported recovery experiences. No researchers were involved in this process. This feedback was based on participants’ responses to each DRAMMA dimension during their last week of vacation before returning to work (T4). The feedback incorporated a social comparison element, categorizing individuals as scoring below, on, or above average. According to the category, tailored recommendations were provided on how to foster recovery behaviors and experiences (for more detail, see availability of data and materials statement). All participants had full access to routine care once included. Each online assessment took approximately 10–30 min to complete. Participants were not financially compensated for their participation.

### 2.4 Eligibility criteria

The inclusion criteria were as follows: (i) downloading the application 14 days prior to their vacation; (ii) over the age of 18; (iii) gainfully employed; (iv) a smartphone with internet access; (v) providing informed consent; (vi) returning to work after their vacation; and (vii) higher levels of work-related rumination, indicated by a score of ≥14 on the Irritation Scale (25). This threshold was selected to enable comparability with prior randomized controlled trials investigating recovery interventions (23, 39). The exclusion criteria were as follows: (i) engaging in other recovery/stress training, (ii) engaging in current psychotherapy, and (iii) consuming variable doses of medication for sleeping complaints.

### 2.5 Intervention content and app design

In short, Holidaily 2.0 was based on Holidaily-app 1.0, which was investigated for usability in a previous study by Smyth et al. (20). While some features were added, such as a guided tour around the app to help explain all available functions, the main components remained unchanged. These include (i) the daily selection of recreational activities called “Dailys” and (ii) the rating after each activity along the six DRAMMA dimensions (22). Dailys are based on behavioral activation strategies that refer to specific activities that positively influence mental health (45). Each Daily corresponds to at least one of the six mechanisms of the DRAMMA-Model. For a full list of all Dailys see availability of data and materials statement. Furthermore, to foster behavioral change, suitable behavior-change techniques, such as health-development tracking and prompts, were included (46). Users also received weekly emails summarizing their recovery profiles to help track their progress. To keep users engaged, a reward system and gamification elements were implemented (47). Points were awarded for completing the Dailys and providing ratings. These points were translated into new features that were displayed on the home screen. A more detailed description can be found in the study protocol (48).

The Holidaily-app consists of four sections (for more detail, see availability of data and materials statement). First, the “Home” screen allows users to track their collected points and provides access to the “Daily” section. Second, the “Daily” section offers activities aligned with different stages of the vacation (before, during, and after). On a daily basis, app users are provided with a random selection of three Dailys to choose from or can create their own recovery activities. Each activity emphasizes one of the DRAMMA mechanisms, varying in intensity, duration, and effort. For goal setting purposes, users answered three questions related to each of the six DRAMMA dimensions. This indicates the users' desired recovery level vs. their current recovery level.

This section is further divided into three areas: (i) Users can “book” a Daily or place it “on hold” for later use. (ii) After completing a Daily, users rate their experience across all six DRAMMA dimensions, fostering reflection on the effectiveness of different activities. Users earn points as a reward for completing Dailys and providing feedback. (iii) An overview of all “on hold”, “booked”, and “done” Dailys was displayed, with search options to locate specific activities.

Third, “Feel” asks users to rate their well-being using three validated one-item questions regarding their experience of their past day, mood, and energy level (49–51). Finally, the “Recovery” section presents users with a summary of their recovery profile, visualized through graphs displaying their recovery and well-being history, as well as individual recovery strengths. This information is based on the data entered by the users. The intention of this section was to promote self-reflection, self-monitoring, and self-efficacy.

Holidaily includes additional practical features. For example, weather forecasts for users' vacation locations, an app walkthrough, and information about the app's scientific background. Holidaily also has a diary function, where users can upload photos and make short notes about how they completed their Daily activities. To personalize the app, users can select one of several avatars (“Holidave” or “Holidaisy”) and select an individual background.

To further increase participants' adherence to the intervention protocol while also reducing nonresponses, as suggested by Newman (52), several aspects were included. For example, users receive push messages as reminders to complete Dailys and rate their well-being. With the sorting function, users can identify Dailys which they rated as particularly useful. On a weekly basis, users received an automated email with a summary of their recovery development. Participants with access to the Holidaily-app were encouraged to complete one Daily per day.

### 2.6 Outcomes

All subjective questions referred to the past seven days unless otherwise stated. An overview of all measures is presented in the results section.

#### 2.6.1 Demographics

Participant demographics, such as age, sex, relationship status, number of children, educational background, work status (part/full-time), work hours per week per contract, actual working hours, length of employment, managerial position, frequency of remote working, last/first workday, and first/last vacation day, were assessed via a self-designed questionnaire two weeks before the vacation.

Vacation specifics, such as the type of vacation, companion, destination, and expectations, were also assessed.

#### 2.6.2 Primary outcome measure

##### 2.6.2.1 Work-related rumination

Work-related rumination was measured using the cognitive irritation subscale of the Irritation Scale (IS) (25). This subscale consists of three items, each with a 7-point Likert response scale (ranging from 0 to 6: lower scores indicate lower levels of work-related rumination) to assess the extent of workers rumination during leisure time, e.g., “Even at home, I often think of my problems at work.”

#### 2.6.3 Secondary outcome measures

##### 2.6.3.1 Mental health

###### 2.6.3.1.1 Depression

The Patient Health Questionnaire (PHQ-8) (53) was used to assess symptoms of depression and consisted of eight items. On a 4-point Likert response scale (ranging from 0 to 3), the respondents indicated how “down, depressed, or hopeless” they felt. A total score of ≥10 indicates clinically significant depression.

###### 2.6.3.1.2 Insomnia severity

Participants' sleep quality was assessed using four items from the Insomnia Severity Index (ISI) (54), and participants were asked to rate their sleep quality on a 5-point Likert scale (ranging from 0 to 4), for example, how much they struggled to sleep during the night. A total score ≥14 indicates clinically relevant insomnia levels (0–7, no insomnia; 8–14, subthreshold insomnia; 15–21, moderate insomnia; and 22–28, severe insomnia).

##### 2.6.3.2 Work-related health

###### 2.6.3.2.1 Thinking about work

Further aspects related to thinking about work were assessed using the Work-Related Rumination Questionnaire (WRRQ) (55). Affective rumination was measured via a subscale consisting of five items and assessing workers' negative and recurring thoughts about work; for example, “Are you troubled by work-related issues when not at work?”. Problem-solving pondering was assessed via a second subscale, also consisting of five items, assessing workers' constructive thoughts, for example “After work I tend to think about how I can improve my performance”. All items were rated on a 7-point Likert response scale (ranging from 0 to 6).

##### 2.6.3.3 Recovery experiences

The DRAMMA questionnaire (DRAMMA-Q), consisting of six subscales measuring detachment, relaxation, autonomy, mastery, meaning, and affiliation, was used to assess participants' recovery experiences. All 18 items were rated on a 5-point Likert scale (range, 0–4). DRAMMA-Q is based on existing validated questionnaires and has been validated (56).

##### 2.6.3.4 Recreational activities

The frequency of participants' engagement in recreational activities after work was assessed using 21 items on a 5-point Likert scale (ranging from 0 to 4) from the Recreation Experience and Activity Questionnaire (ReaQ) (57).

##### 2.6.3.5 Emotional exhaustion

The participants' emotional exhaustion was measured using five items from the exhaustion subscale of the Maslach Burnout Inventory-General Survey (MBI-GS-D) on a 6-point Likert scale (ranging from 0 to 6) (58). The participants rated statements indicating whether their work made them feel emotionally drained or exhausted.

#### 2.6.4 Tertiary variables

##### 2.6.4.1 Work environment

Several work characteristics of participants were assessed on a scale of 1–5, i.e., the number of unfinished tasks (59), time pressure (ISTA) (60), autonomy concerning the order in which tasks were completed and the variety of tasks (WDQ) (61), and social support from colleagues (SzSU) (62).

#### 2.6.5 Work non-work interface

To assess participants' ability to cope with boundaries between work and private life, the Work-life Indicator (WLI) (63), the Work-Life-Balance scale (on a scale of 1–6) (TKS-WLB) (64) (on a scale of 1–6), and the Life Satisfaction Scale (SWLS) (65) were used.

#### 2.6.6 Well-being

Further traits concerning participants’ well-being were assessed with measures such as vitality (PANAS) on a scale of 1–7 (66, 67), perceived need for recovery (NFR) on a scale of 1–5 (68), physical health and well-being, for example (69), and resilience (RS) on a scale of 1–5 (70).

#### 2.6.7 Performance

The following measures were used to assess participants' performance: work engagement (UWES) on a scale ranging from 1 to 7 (71), objective and subjective creativity (TTCT) on scales ranging from 1 to 5 (72–74), and work performance (OCB) on a scale ranging from 1 to 7 (75).

#### 2.6.8 User experience

The participants' digital experiences via Holidaily were assessed using a 28-item questionnaire from AttrakDiff2 (76). A seven-point Likert scale was used for the responses (1–7). The questionnaire provides information on global attractiveness (ATT), pragmatic quality (PQ), hedonic quality-identity (HQ-I), and hedonic quality-stimulation (HQ-S). Scores below four indicate a negative trend, and scores above four indicate positive user experience.

### 2.7 Sample size calculation

The sample size calculation was based on the expected standardized difference in the primary outcome measure, work-related rumination (Irritation Scale) (25), at two weeks after vacation. Despite meta-analyses and RCTs indicating promising evidence for standalone mental health apps in clinical samples and for mobile interventions addressing stress among workers, their efficacy remains unclear (77–79).

Furthermore, from a public health perspective, even small effect sizes may be meaningful when interventions are scalable and aimed at the general working population, as they have the potential to reach a broader audience. Considering these two lines of reasoning, we regard an effect size of *d* = 0.20 for work-related rumination as meaningful, especially when considering that the app represents a low-intensity, self-help intervention for the general working population. *A priori* G*Power analysis (80) for a two-tailed test, in which 80% power and a 5% significance level were assumed, indicated that an overall sample size of *N* = 788 was needed to detect significant differences between groups.

### 2.8 Randomization

The participants were randomly allocated in blocks with variable block lengths at a 1:1 ratio to the intervention or control group, ensuring an equal distribution of participants into both groups. The concealed allocation sequence for randomization was automatically conducted through the online platform Unipark (Questback, Cologne, Germany) and thus did not involve the researchers. Blinding of the participants and coaches was not feasible.

### 2.9 Statistical methods

All results were based on the intention-to-treat principle (ITT), and analyses were performed with SPSS, version 21 (IBM Corp, Armonk. NY, USA). ITT analyses include all randomized participants, irrespective of protocol deviations, withdrawals, or non-completion of questionnaires (81). Analyses were reported according to the Consolidated Standards of Reporting Trials (CONSORT) for web-based and mobile health interventions (42). All reported *p-values* were two-sided, with a significance level of *α* = 0.05. Descriptive statistics were used to analyze sample characteristics.

#### 2.9.1 Missing data

All participants completed the baseline assessment. To handle missing values, a Markov chain Monte Carlo multivariate imputation algorithm (SPSS 21) with 100 estimations per missing value was used to estimate the values at T2, T3, T4, T5, and T6 (82). In the multiple imputation approach (MI), predictors are defined to generate plausible estimations for missing values.

#### 2.9.2 Primary analysis

Analyses of covariance (ANCOVAs) were conducted for the primary and all continuous secondary outcomes. Baseline scores were included as covariates to control for differences and increase statistical power. ANCOVA was used to detect between-group differences and is considered an ideal statistical method for the analysis of continuous outcomes in RCTs in terms of bias, precision, and statistical power (83, 84). Cohen's *d* was calculated on the basis of group differences in marginal means of the ANCOVA post-vacation, standardized by the pooled standard deviation of the post-vacation scores (85).

#### 2.9.3 Sensitivity analyses

To verify the robustness of the findings, several sensitivity analyses were conducted. First, ANCOVAs were conducted with the study completer sample for the primary and secondary outcomes. The completer analysis refers to the process of repeating the same ANCOVA but restricting the dataset to participants who provide a full set of follow-up data; thus, no imputation is needed.

Second, a further sensitivity analysis was employed as a linear mixed-effects model to examine changes in work-related rumination over time (T1–T5) between the intervention and waitlist control groups.

### 2.10 Explorative analysis

#### 2.10.1 Description of change

Changes in scores relative to the respective two weeks prior to vacation (baseline) levels were calculated for all measurement time points to indicate development and ideally quantify improvements found for participants. Two-sided *t*-tests were conducted to test the significance of changes compared with the baseline values.

### 2.11 Ethical approval

All procedures were approved by the Ethics Committee of Leuphana University in Lueneburg (reference number: 201606, EB-Antrag Lehr201606_holidaily), and the trial was registered at the German Clinical Trials Registry (DRKS-ID: DRKS00013650, January 15, 2018). Informed consent was obtained from all participants prior to the study.

## 3 Results

### 3.1 Sample description

**Figure 1:**
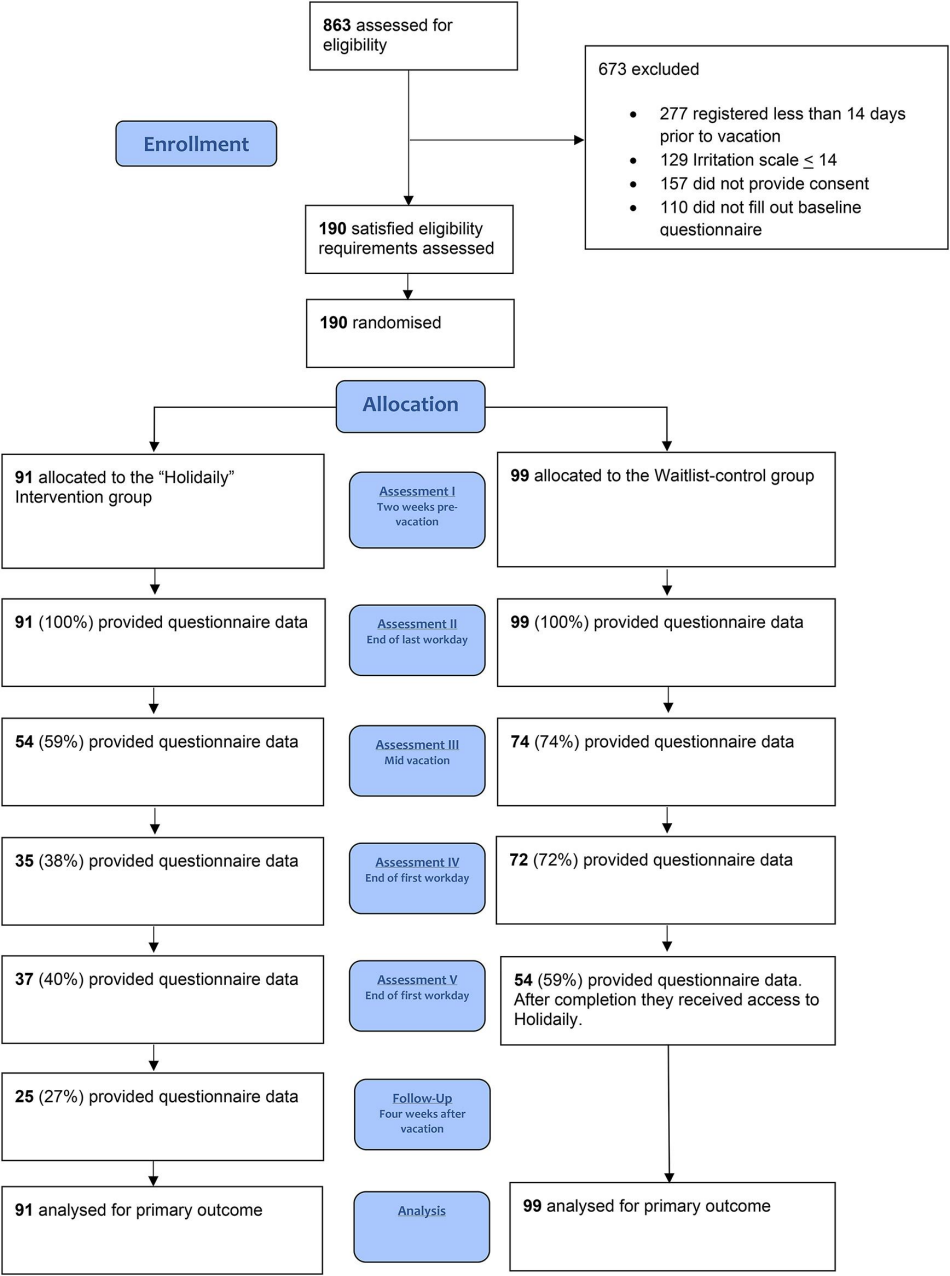
Flow of participants.

Figure 1 depicts the study flowchart, and Table 1 summarizes the detailed baseline characteristics of the study participants. The sample consisted of 190 workers, most of whom were female (151/190, 79%), on average 33 years old (*SD* = 10.6), married or in a relationship (152/190, 80%), and attended higher education (121/190, 64%). The majority of the participants were employed full-time (158/190, 83%), had permanent contracts (158/190, 83%), and had 15 years (*SD* = 9.1, range: 0–46 years) of occupational experience.

**Table 1:** Demographic characteristics: means/outcomes, standard deviations/percentages at baseline.

		Total (*N* = 190)		Holidaily (*n* = 91)		Control (*n* = 99)	
Baseline characteristic		N/m	%/SD	n/M	%/SD	n/M	%/SD
Age (*M/SD*)		33.3	11.0	33.3	11.3	22.3	10.0
Gender	Male	39	21	20	22	19	19
	Female	151	80	71	78	80	81
Relationship	Married or in a partnership	152	80	71	78.1	81	82
Education	Low	31	17	14	15	18	18
	Medium	30	16	11	12	19	19
	High (University)	121	67	64	70	57	58
Work Characteristics	Work experience in years (*M/SD*)	15.2	9.0	16.4	10.0	14.1	9.0
	Employed fulltime	150	79	74	81	76	77
	Work experience more than 5 years	114	60	59	65	55	56
	Temporary employment	23	12	10	11	13	13
	Permanent employment	158	83	78	86	80	81
	Self-employed	9	5	3	3	6	6
Mental health	Leadership position	68	36	28	31	40	40
	No depression^a^	91	48	58	64	33	33
	Screening of depression^b^	71	49	33	36	38	38
	No or subclinical insomnia^c^	127	67	58	64	69	76
	Moderate or severe insomnia^d^	63	33	33	36	30	33
	Overall life satisfaction (*M/SD*)	3	1	3	1	3	1

a PHQ- score of 0–9 (53).

b PHQ- score of ≥10.

c Insomnia severity index of 0–7 (54).

d Insomnia severity index of ≥8.

Notably, 71% (135/190) of the participants scored above 18 on the PHQ-8, indicating that a substantial proportion experienced elevated symptoms of depression. Among those with sleep difficulties, 33% (63/190) experienced subthreshold symptoms.

Data were missing for 41% of the participants in the intervention group and 26% of those in the control group at T2, and for 62% and 26% at T3, respectively (Figure 1).

Table 2 shows the means and standard deviations for all outcomes and measurement time points. With respect to the six recovery experiences assessed by the DRAMMA questionnaire, the scores were, on average, slightly below the midpoint of the scale, indicating a medium level, except for that of affiliation. For affiliation, participants appeared to experience a relatively high level of connectedness, mirrored by the above midpoint score, even before the vacation.

**Table 2:** Means and standard deviations of the primary and secondary outcome variables.

	T1				T2				T3				T4				T5				T6	
Outcomes	Holidaily		Control		Holidaily		Control		Holidaily		Control		Holidaily		Control		Holidaily		Control		Holidaily	
	M	SD	M	SD	M	SD	M	SD	M	SD	M	SD	M	SD	M	SD	M	SD	M	SD	M	SD
Primary Outcome																						
Work-related rumination	16.1	3.6	15.9	3.5	15.5	2.7	15.6	3.1	10.6	3.7	11.2	4.3	12.8	2.7	12.4	3.6	12.5	3.2	14.8	4.0	11.9	2.4
Secondary Outcomes																						
*Mental health*																						
Depression	8.3	4.7	8.3	4.1	–	–	–	–	–	–	–	–	4.6	2.7	4.1	3.5	6.5	3.1	6.8	3.8	6.6	2.6
Insomnia Severity	7.4	3.1	7.1	2.9	7.3	2.2	7.4	2.6	6.1	2.1	6.1	2.4	5.9	1.8	6.0	2.3	6.1	1.8	6.6	2.2	–	–
*Work-related health*																						
Affective rumination	22.1	5.9	21.0	6.4	18.9	6.6	20.1	7.0	13.0	5.9	14.0	6.8	12.2	5.2	12.6	5.8	13.8	5.1	15.2	6.0	–	–
Problem-solving pondering	16.7	6.7	18.7	7.4	21.0	4.5	21.4	5.5	11.0	3.8	12.0	5.2	10.1	3.5	11.0	4.6	13.8	4.2	15.8	5.8	–	–
Recovery Experiences	3.0	0.6	3.1	0.5	2.9	0.5	2.8	0.5	3.8	0.4	3.7	0.5	3.8	0.4	3.8	0.4	3.4	0.4	3.4	0.4	3.4	0.3
Detachment	2.5	0.6	3.0	0.5	2.9	0.5	2.8	0.5	3.8	0.6	3.7	0.8	3.8	0.5	3.8	0.6	3.2	0.6	3.2	0.6	3.3	0.4
Relaxation	2.7	0.8	2.6	0.8	2.5	0.7	2.3	0.8	3.9	0.6	3.7	0.7	3.9	0.5	3.9	0.7	3.4	0.6	3.2	0.7	3.3	0.5
Autonomy	3.1	0.9	3.2	0.9	3.0	0.6	2.9	0.7	4.0	0.6	3.8	0.6	3.9	0.6	3.9	0.6	3.4	0.6	3.5	0.7	4.6	0.4
Mastery	2.8	1.2	2.8	1.0	2.7	0.7	2.7	0.8	3.4	0.7	3.3	0.8	3.4	0.7	3.3	0.9	3.1	0.7	3.2	0.8	2.9	0.5
Meaning	2.9	1.0	2.9	1.0	2.9	0.8	2.8	0.8	3.5	0.7	3.4	0.8	3.5	0.6	3.4	0.8	4.4	0.7	3.2	0.8	3.1	0.5
Affiliation	3.8	0.7	3.9	0.6	3.7	0.5	3.8	0.6	4.3	0.4	4.3	0.6	4.3	0.4	4.3	0.5	4.0	0.5	4.0	0.5	4.1	0.4
Recreational activities	26.6	10.9	23.6	10.1	–	–	–	–	–	–	–	–	–	–	–	–	28.4	6.9	27.9	7.9	32.9	7.3
Emot. Exhaustion	23.3	5.7	6.8	6.8	–	–	–	–	–	–	–	–	17.7	5.8	17.8	5.9	18.9	5.6	18.3	5.7	17.5	4.2

Holidaily: *n* = 91, Control: *n* = 99; variables assessed at baseline two weeks pre-vacation (T1), end of last workday (T2), mid-vacation (T3), end of first workday (T4), two weeks post-vacation (T5) and four weeks post-vacation (T6, only intervention group), M, mean; SD, standard deviation.

On average, the participants were on vacation for 16 days. The majority travelled with their significant other (81/190, 43%) or alone (74/190, 39%), labelled their type of vacation as relaxing (114/190, 60%), and chose Germany (52/190, 27%) as their destination (Table 3).

**Table 3:** Overview of the vacation characteristics.

		Total (*N* = 190)		Holidaily (*n* = 91)		Control (*n* = 99)	
Vacation characteristics		N/M	%/SD	n/M	%/SD	n/M	%/SD
Lenth of vacation (*M/SD*)		16.4	6.7	16.3	7.0	16.4	6.50
Person vacation spent with	Significant other	81	43	32	35	49	49
	Children	18	9	7	8	11	11
	Family	22	12	13	14	9	9
	Extended Family	6	3	3	3	3	3
	Friends	22	12	6	7	16	16
	Colleagues	2	1	1	1	1	1
	Acquaintances	1	0.5	1	1	–	–
	Alone	74	39	32	35	42	42
Type of vacation	Staycation	26	14	13	14	13	13
	Relaxation	114	60	46	51	68	68
	Roundtrip	25	13	10	11	15	15
	Activity	40	21	17	19	23	23
	Culture	14	7	1	1	13	13
	Nature	35	18	20	22	15	15
	City	28	15	8	9	20	20
Vacation destination	Visiting family	18	9	7	8	11	11
	Germany	52	27	25	27	27	27
	Spain	15	8	5	5	10	11
	Portugal	6	3	4	4	2	2
	Scandinavia	8	4	2	2	6	6
	Greece	4	2	2	2	2	2
	Croatia	2	1	2	2	–	–
	Italy	3	2	1	1	2	2
	South Africa	2	1	1	1	1	1
	Turkey	2	1	1	1	1	1
	Austria	4	2	1	1	3	3
	Thailand	2	1	1	1	1	1
	Australia	1	0.5	–	–	1	1
	US	5	3	2	2	3	3

### 3.2 Primary outcome analysis

#### 3.2.1 Work-related rumination two weeks post-vacation

**Figure 2:**
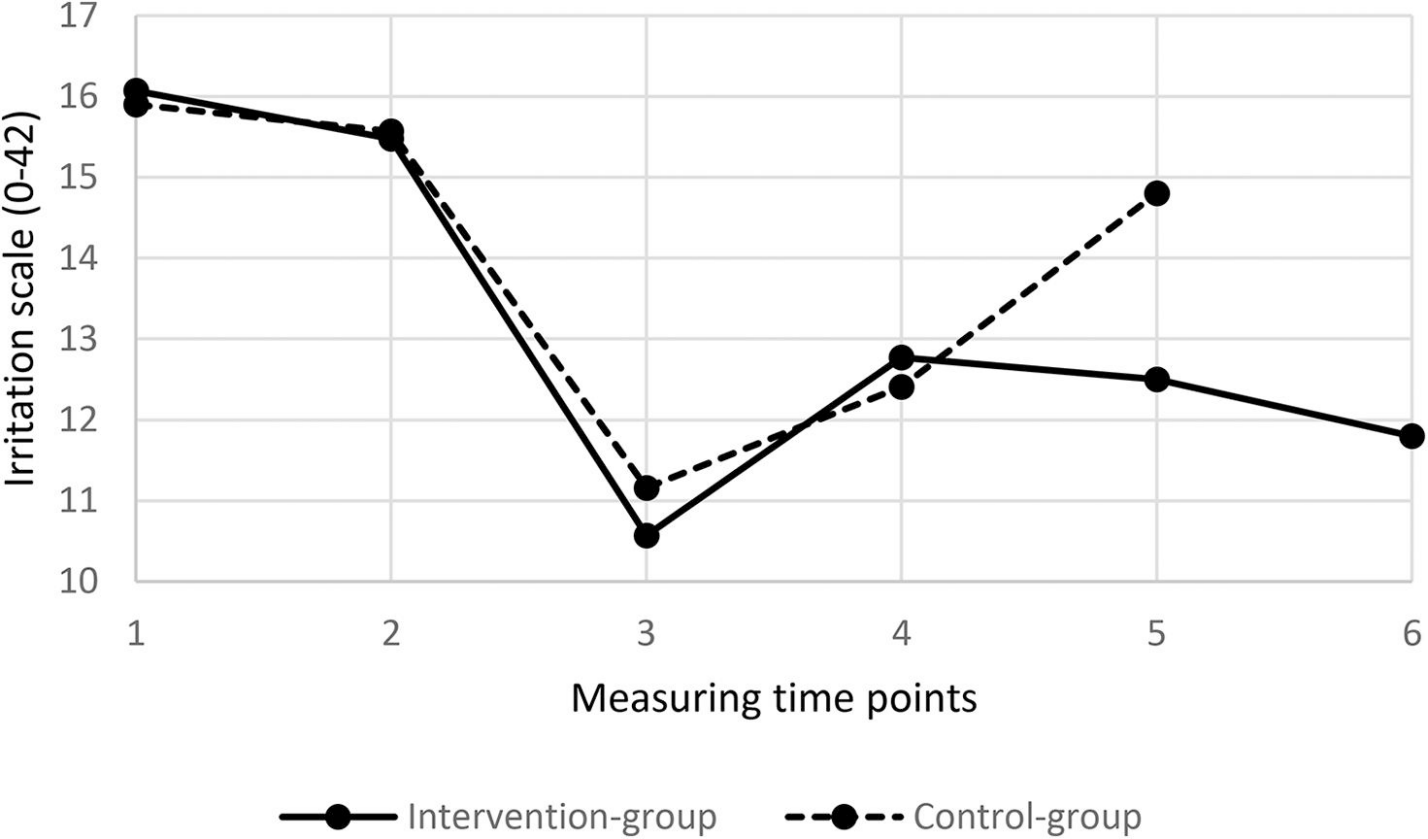
Work-related rumination across different time measurement points. The work-related rumination rate was measured on an irritation scale in the intention-to-treat sample. The time points used were as follows: 1 = baseline at two weeks pre-vacation, 2 = end of the last workday, 3 = mid-vacation, 4 = end of the first workday, 5 = two weeks post-vacation, and 6 = four weeks post-vacation, measured only in the intervention group. Lower scores indicate reduced work-related rumination.

The ANCOVA for the primary outcome, controlling for baseline scores, revealed a significant difference between groups in favor of the intervention group at two weeks post-vacation: F(_1,187_) = 23.81, *p* < .001, resulting in an effect of *d* = −0.67 (95% CI −1.0; −0.4), corresponding to a Δ = 2.3 in work-related rumination. Compared with the waitlist control condition, the intervention group experienced a greater effect on work-related rumination two weeks after resuming their daily work lives (Table 4). Figure 2 shows work-related rumination scores across all six measurement time points for the intervention and control group. Homogeneity of error variances using Levene's test was conducted. Results suggested no evidence of heteroscedasticity [F(_1, 188_) = 2.213, *p* = .139].

**Table 4:** Between-group differences at 2 weeks post-intervention (T5) in the intention-to-treat and study completer sample.

Outcomes	Between-groups effect T5 Intention-to treat (*N* = 190)				Between-groups effect T5, Study completer (*n* = 91)			
	Cohens’ *d*	95% CI	*F*	*p* value	Cohens’ *d*	95% CI	*F*	*p* value
Primary outcome								
Work-related rumination	−0.67	−1.0; −0.4	22.9	<.001	−0.50	−0.1; −0.9	8.2	.005
Secondary outcomes								
*Mental health*								
Depression	0.02	−0.3; 0.3	0.2	.684	−0.05	−0.5;0.3	0.7	.744
Insomnia Severity	−0.35	−0.6;−0.1	5.8	.030	−0.38	−0.8;0.1	3.1	.080
*Work-related health*								
Affective Rumination	−0.27	−0.6; −0.1	8.7	.040	−0.30	−0.7; −0.1	9.0	.040
Problem-solving pondering	−0.34	−0.7; −0.1	7.6	.006	−0.41	−0.9;0.0	3.7	.058
Recovery Experiences	0.11	−0.2;0.4	0.8	.355	0.09	−0.3;0.5	1.8	.181
Detachment	−0.01	−0.4;0.2	1.2	.267	0.05	−0.4;0.5	0.8	.370
Relaxation	0.13	−0.2;0.4	2.5	.111	0.32	−0.1;0.7	2.6	.110
Autonomy	−0.04	−0.3;0.2	0.1	.774	−0.23	−0.6;0.2	1.6	.213
Mastery	−0.03	−0.3;0.3	0.1	.775	−0.14	−0.5;0.3	0.3	.603
Meaning	0.08	−0.2;0.3	0.3	.613	0.07	−0.3;0.5	0.4	.557
Affiliation	0.03	−0.3;0.3	0.2	.684	0.09	−0.3;0.5	1.5	.223
Recreational activities	−0.10	−0.4;0.2	0.5	.498	−0.02	−0.5;0.4	0.01	.925
Emot. Exhaustion	−0.06	−0.4;0.2	0.1	.708	−0.25	−0.7;0.2	1.2	.274

#### 3.2.2 Sensitivity analyses

Several sensitivity analyses were conducted to verify the robustness of the main effect. First, an ANCOVA was performed for the study completer sample (Table 4). The results revealed a significant difference between groups [F(_1,104_) = 22.93, *p* < .005] in favor of the intervention group at two weeks post-vacation, with an effect size of *d* = 0.50 (95% CI −0.1; −0.9) for work-related rumination.

Second, the mixed model analysis revealed a significant interaction effect between group and time (*β* = 2.46, 95% CI 1.31–3.62, *t* = 4.18).

At baseline (T1), both groups presented comparable levels of work-related rumination (intervention: *M* = 16.07, *SD* = 3.55; waitlist control: *M* = 15.90, *SD* = 3.5; *p* = .75). However, at follow-up (T5), the intervention group demonstrated significantly lower work-related rumination scores (*M* = 12.49, *SD* = 3.17) than the waitlist control group (*M* = 14.79, *SD* = 3.95; *p* < .0001), with an estimated difference of 2.30 points.

Separate within-group analyses revealed that the intervention group exhibited a substantial and statistically significant reduction in work-related rumination from T1 to T5 [Δ =  −3.57, 95% (CI −4.43; −2.70), *p* < .0001], whereas the control group showed a smaller yet still significant decrease (Δ = −1.10, 95% CI −1.88; −0.32, *p* = .006) in work-related rumination.

#### 3.2.3 Explorative analyses

For the exploratory analyses, vacation effects were operationalized as within-person change from baseline by calculating change scores (follow-up minus baseline) at each assessment.

#### 3.2.4 Description of change

Changes in work-related rumination relative to the baseline (two weeks prior to vacation) were evaluated to quantify improvements found for participants over time (Table 5). During the pre-vacation (T1–T2) period, from two weeks to the last working day, the scores remained almost stable within each group. At mid-vacation (T1–T3), work-related rumination declined largely by 34% in the intervention group and 30% in the waitlist control group, indicating a robust vacation effect. On the first working day post-vacation, a substantial reduction compared with pre-vacation (T1–T4) was observed in both groups: 21% in the intervention group and 22% in the control group. Similarly, two weeks after returning to work (T1–T5), both groups showed a beneficial effect compared to the respective baseline levels. However, while the improvement in the control group was 7% greater, the beneficial effect in the intervention group was still 22%, which further increased to 26% at four weeks post-vacation (T1–T6).

**Table 5:** Percentages of change from baseline of primary and secondary outcome variables, intention-to-treat sample (*N* = 190).

Outcomes	Prevacation effect (T1–T2)				Mid-vacation-effect (T1–T3)				End-of-vacation-effect (T1–T4)				Two-weeks-fade-out-effect (T1–T5)				Four-weeks-fade-out-effect (T1–T6)	
	Holidaily		Control		Holidaily		Control		Holidaily		Control		Holidaily		Control		Holidaily	
	%	*p* value	%	*p* value	%	*p* value	%	*p* value	%	*p* value	%	*p* value	%	*p* value	%	*p* value	%	*p* value
Primary Outcome																		
Work-related rumination	3.7	.515	2.1	<.001	34.2	<.001	29.8	<.001	20.5	.001	22.0	.005	22.2	<.001	6.9	.250	26.1	.005
Secondary Outcomes																		
*Mental health*																		
Depression	–	–	–	–	–	–	–	–	45.1	<.001	44.5	<.001	21.8	<.001	20.9	.016	20.9	.012
Insomnia Severity	7.1	.697	4.9	.001	17.5	.010	14.4	.003	19.8	.022	15.1	.003	17.1	.005	6.3	.005	–	–
*Work-related health*																		
Affective rumination	14.5	.174	10.0	.274	41.3	<.001	33.6	<.001	44.7	<.001	39.8	<.001	37.7	<.001	26.1	<.001	–	–
Problem-solving pondering	25.4	.111	14.3	.111	34.4	<.001	35.8	<.001	39.7	<.001	41.6	<.001	17.6	<.001	17.1	<.001	–	–
Recovery Experiences	3.2	.460	6.7	<.001	28.9	<.001	23.2	<.001	28.7	<.001	26.0	<.001	14.8	<.001	12.1	.500	13.7	.001
Detachment	3.7	.752	14.4	<.001	52.8	<.001	33.7	<.001	52.9	<.001	36.7	<.001	28.9	<.001	17.0	.205	31.9	.005
Relaxation	7.6	.486	10.6	<.001	43.6	<.001	43.6	<.001	46.5	<.001	51.5	<.001	24.9	<.001	22.6	<.001	22.3	.004
Autonomy	3.1	.169	7.5	.007	28.6	<.001	21.6	<.001	25.3	<.001	24.7	<.001	10.5	.007	9.4	.039	14.0	.003
Mastery	3.5	.500	4.1	.609	22.5	<.001	19.9	<.001	22.3	<.001	20.7	<.001	11.9	.082	13.8	.003	3.0	.185
Meaning	2.1	.259	0.1	.729	24.2	<.001	19.8	<.001	22.9	<.001	20.8	<.001	15.4	.010	14.4	.023	10.1	.152
Affiliation	3.4	.669	4.6	.013	11.2	<.001	8.4	<.001	11.8	<.001	13.3	<.001	3.5	.004	0.5	.597	6.1	.012
Emot. Exhaustion	–	–	–	–	–	–	–	–	24.2	<.001	16.5	<.001	19.0	.002	12.9	<.001	25.1	<.001

Holidaily: *N* = 91, Control: *N* = 99; variables assessed at baseline two weeks pre-vacation (T1), end of last workday (T2), mid-vacation (T3), end of first workday (T4), two weeks post-vacation (T5) and four weeks post-vacation (T6, only intervention group), % = percentage of change from baseline; *p*-value of paired two-sided *t*-tests.

### 3.3 Secondary outcome analyses

#### 3.3.1 Differences between groups at two weeks post-vacation

We investigated the beneficial effects of the intervention on all secondary outcomes by conducting further ANCOVAs for the ITT sample two weeks post-vacation (Table 4).

For mental health outcomes, no significant group differences were found for depression (*p* = .684). However, for sleep a significant effect was observed [F(_1,187_) = 5.84, *p* = .030, *d* = −0.35 (95% CI −0.06; −0.1)].

Regarding work-related outcomes, additional analyses were conducted using subscales of the Work-Related Rumination Questionnaire (WRRQ) (26), which assesses different forms of work-related rumination. Specifically, affective rumination reflects negative, recurrent thoughts about work, whereas problem-solving pondering captures more constructive, work-related perseverative thinking. Significant effects in favor of the intervention group were observed for affective rumination [F(_1,187_) = 8.74, *p* = .04, *d* = −0.27 (95% CI −0.06;−0.1), Δ = 1.48] and problem-solving pondering [F(_1,187_) = 7.62, *p* = .006, *d* = −0.34 (95% CI −0.7; −0.18), Δ = 2.00]. A similar pattern was observed in the study completer sample for affective rumination [F(_1,85_) = 9.02, *p* = .04, *d* = −0.30 (95% CI −0.7;.0.1)], but not for problem-solving pondering (*p* = .058).

No significant group differences were found for recovery experience assessed with the DRAMMA-questionnaire (detachment *p* = .267; relaxation *p* = .111; autonomy *p* = .774; mastery *p* = .775; meaning *p* = .613; affiliation *p* = .684). Likewise, no significant effects were observed for recreational activities (*p* = .498) or emotional exhaustion (*p* = .708).

#### 3.3.2 Description of change

Additionally, the effects of vacations on mental health (depressive symptoms, sleep quality), and work-related health (affective rumination, problem-solving pondering, recovery experiences, and emotional exhaustion) were evaluated (Table 5). Because recreational activities were assessed at only three measurement time points (T1, T5 & T6), they were not included in the analyses of change.

For depressive symptoms, a substantial reduction between the pre-vacation phase and the first working day after vacation (T1–T4) was observed in both groups, with symptoms declining by approximately 45% in both the intervention and the control group. Two weeks after returning to work (T1–T5), depressive symptoms remained lower than baseline levels, with reductions of 22% in the intervention group and 21% in the control group. For the intervention group, this improvement was sustained even four weeks post-vacation (T1–T6), with a reduction of 21%.

A similar pattern was observed for sleep quality. Both groups reported improved sleep during the vacation period. These improvements gradually declined after returning to work but remained slightly better than baseline levels in both groups.

Similar temporal patterns were observed for affective rumination and problem-solving pondering. Both constructs decreased during the vacation period and increased again after returning to work, although levels remained slightly below baseline in the intervention group.

Regarding recovery experiences, a slight decline during the pre-vacation phase was observed, likely reflecting the completion of work-related tasks and vacation preparations. The mid-vacation effect (T1–T3) for recovery experiences ranged from 22% to 53% among app users and from 20% to 44% among controls, except for the experience of affiliation, where baseline scores were already very high. At the end of vacation (T1–T4), recovery experiences remained elevated in both groups. Although these effects declined two weeks after returning to work in both groups (T1–T5), recovery levels were still 10%–30% higher in the intervention group and 9%–23% higher in the control group compared with baseline, again with little change in affiliation.

Emotional exhaustion followed a pattern similar to sleep quality. Participants in both groups reported lower exhaustion during the vacation period, with levels gradually increasing again after returning to work but remaining slightly below baseline levels.

### 3.4 User experience and satisfaction

User experience, as indicated by attractiveness, pragmatic quality (useability), and hedonic qualities of identity and stimulation, ranged from 4.8 to 5.1, and thus was rated above the neutral mean of 4 on the respective bipolar scales (Table 6). Two weeks after the vacation, 66% of the intervention group would recommend the Holidaily-app to a friend, and 69% intended to continue using the app. The participants' feedback regarding the intervention was positive. Responses to an open-ended question indicated high levels of satisfaction and joy, with the only recurring criticism being the length of the questionnaires.

**Table 6:** Means and standard deviations of user experience and user satisfaction for the intervention group.

Outcomes	*n*	M	SD	%
App user experience				
Attractiveness (ATT)	36	5.06	1.16	
Pragmatic quality (PQ)	36	4.57	1.18	
Hedonic quality-identity (HQ-I)	36	4.59	0.97	
Hedonic quality-stimulation (HQ-S)	36	4.81	0.99	
App user satisfaction				
continued usage	32			66
recommend Holidaily	32			69

Global attractiveness (ATT), pragmatic quality (PQ), hedonic quality-identity (HQ-I), and hedonic quality-stimulation (HQ-S) were assessed on a 7-likert scale (range: 1–7).

### 3.5 Tertiary outcome analysis

Table 7 shows the results of the ANCOVA for the ITT and completer samples for all tertiary outcomes. In terms of the ITT analyses, significant differences between groups and in favor of the intervention group were found for time pressure, need for recovery, and creativity. However, when the same ANCOVA was repeated with completers only, these findings could not be confirmed, suggesting that ITT effects are sensitive to assumptions about missing data and should be interpreted cautiously.

**Table 7:** Means and standard deviations of tertiary outcome variables.

Outcomes	T1				T2				T3				T4				T5				T6	
	Holidaily		Control		Holidaily		Control		Holidaily		Control		Holidaily		Control		Holidaily		Control		Holidaily	
	M	SD	M	SD	M	SD	M	SD	M	SD	M	SD	M	SD	M	SD	M	SD	M	SD	M	SD
Tertiary Outcomes																						
Work Environment																						
Unfinished Tasks	2.8	1.0	2.9	1.1	2.3	0.9	2.6	1.0	–	–	–	–	2.6	0.7	3.0	1.0	2.6	0.7	2.7	0.7	–	–
Time Pressure	3.1	1.0	3.1	1.0	3.2	0.8	3.4	1.0	–	–	–	–	2.1	0.5	2.5	0.9	2.8	0.7	3.0	0.7	–	–
Task Variety	3.8	1.0	4.1	0.8	–	–	–	–	–	–	–	–	–	–	–	–	–	–	–	–	–	–
Social Support at work	3.2	0.9	3.1	0.9	–	–	–	–	–	–	–	–	–	–	–	–	–	–	–	–	–	–
Work/non-work interface																						
Boundary management	3.1	0.8	3.0	0.9	–	–	–	–	–	–	–	–	–	–	–	–	–	–	–	–	–	–
Work-Life Balance	3.2	1.1	3.1	1.1	2.8	0.8	2.8	0.9	–	–	–	–	–	–	–	–	3.5	0.8	3.6	0.9	3.9	0.6
Wellbeing																						
Resilience	17.3	3.6	18.0	3.4	–	–	–	–	–	–	–	–	–	–	–	–	18.6	1.8	19.1	2.3	–	–
Vitality	14.0	4.5	14.7	4.3	15.3	4.0	14.3	3.7	18.4	3.1	18.4	4.5	17.4	3.9	17.9	4.1	16.7	3.6	16.9	3.5	16.2	2.9
Physical Health	2.5	1.1	2.4	1.2	–	–	–	–	–	–	–	–	–	–	–	–	–	–	–	–	–	–
Need for Recovery	14.4	3.1	13.9	3.4	14.6	2.5	15.0	3.0	–	–	–	–	10.7	3.0	10.3	3.2	12.0	2.4	12.5	3.0	10.8	1.9
Performance																						
Work Engagement	3.7	1.4	3.8	1.2	3.7	1.1	3.9	1.0	–	–	–	–	3.9	1.0	4.1	1.1	3.9	0.9	4.0	1.0	4.0	0.7
Work Performance	5.1	0.9	5.1	0.8	5.1	0.7	5.3	0.7	–	–	–	–	5.1	0.6	5.0	0.7	5.1	0.5	5.0	0.6	–	–
Creativity	2.5	1.0	2.6	1.0	2.4	0.8	2.6	0.9	–	–	–	–	2.5	0.6	2,4	0.9	2.5	0.5	2.7	0.7	–	–

No harms of the intervention were reported; however, these were not systematically monitored with a questionnaire. Yet it was examined whether participants showed a relative deterioration of >20% on the primary outcome (86). Using this criterion, four participants met the threshold for worsening (three in the control group and one in the intervention group).

## 4 Discussion

The aim of this study was to examine the efficacy of the gamified smartphone-based intervention Holidaily 2.0 in promoting recovery behavior with the aim of prolonging the beneficial effect of vacation among workers in a randomized controlled trial. Two weeks after returning to work, participants in the intervention group reported significantly lower levels of work-related rumination (*d* = 0.67) than the waitlist control group, who received automatically generated, personalized feedback. Several sensitivity analyses confirmed this finding. Compared to their levels of work-related rumination two weeks before vacation, the control group showed 6.9% reduction two weeks after vacation, while the intervention group showed a 22.2% reduction, which persisted for up to four weeks after returning to work (26.1%). These findings suggest that a low-threshold digital intervention can prolong the beneficial effect of vacation on work-related rumination.

The results from an alternative, empirically highly correlated measure (27) of work-related rumination, namely affective rumination (55), supported this finding. They indicate a significant reduction in negative, intrusive and recurring thoughts about work in the intervention group. The results from both measurement instruments consistently imply that the intervention has a positive effect on a core feature of the recovery process (87). Similar to work-related and affective rumination, problem-solving pondering establishes a mental connection with work or work-related stressors but in contrast focuses on finding ways to improve and solve problems (87). Although the intervention had a positive effect on problem-solving pondering in the intention-to-treat sample, indicating a positive mental connection to work as opposed to being “switched off,” this effect was not replicated in the sample of study completers. Interestingly, a recent study found that affective rumination had a stronger predictive impact on mental health than problem-solving, pondering, or mental detachment (88). For the remaining secondary outcomes, no consistent statistically significant group differences were observed. Although insomnia severity showed a significant effect in the intention-to-treat analysis, this effect was not replicated in the completer sample. Overall, the findings therefore suggest that the intervention effects were primarily confined to the reduction of work-related, negative, repetitive thinking that is associated with a negative affective state. Due to the limitation of the sample size and multiple testing the results for secondary outcomes should be interpreted exploratively.

These patterns imply that targeting work-related rumination specifically, rather than broader recovery constructs, may represent a particularly efficient intervention pathway for sustaining post-vacation wellbeing. Although previous studies have reported improved recovery scores post-vacation compared with pre-vacation (7, 19, 20), the role of a mobile intervention in sustaining the beneficial effect has remained unknown thus far, as studies have not included control groups. The inclusion of a control group in the present study provides novel insights but simultaneously limits the direct comparability with prior studies.

In terms of work-related rumination, the present study revealed a larger between-group effect size than expected *a priori* in the sample size calculation (*d* = 0.67). By comparison, the effect is larger than the findings of the subgroup analysis for work-related rumination in Karabinski et al.'s (2) meta-analysis (*d* = 0.35). The larger effect on work-related rumination may be explained by differences in intervention focus. The Holidaily-app specifically targeted the reduction in work-related rumination, which was not reflected in the primary studies included in Karabinski et al.'s (2) subgroup analysis. Importantly, none of the mobile interventions included in Karabinski's (2) meta-analysis targeted rumination directly, which overall makes drawing comparisons difficult. Instead, three RCTs included by Karabinski, evaluating a web-based recovery training, reported larger effects on work-related rumination, using the same measurement method as in the present study. In a sample of 128 teachers with elevated rumination, Ebert et al. (23) found a large effect for the self-guided version compared with a waitlist control (*d* = 1.06). In a similar trial with 177 workers without symptomatic inclusion criteria, Behrendt et al. (38) observed a similarly large effect for an unguided recovery training relative to waitlist (*d* = 0.87). Thiart et al. (39) studied 128 teachers with clinical insomnia and elevated rumination and reported a large effect for a guided recovery training compared with waitlist controls (*d* = 0.73). Direct comparisons with the present findings are constrained, as these programs focused on restorative sleep, were more intensive and, in some cases, including personalized feedback and guidance from digital coaches. Nonetheless, the pattern aligns with broader evidence suggesting that fully self-guided mobile interventions tend to yield smaller individual-level effects than more intensive web-based programs, while still offering potential public health value if implemented successfully at scale in the general working population (40).

In contrast to previous studies on digital interventions (36, 37, 89), the present study found improvements in work-related rumination, but not in psychological detachment. This pattern differs from earlier findings, such as those reported by Karabinski et al. (2), who found nearly identical effects for reductions in negative rumination and increases in detachment. In the present study, although work-related rumination decreased significantly, psychological detachment showed only a descriptive positive trend, with a 28.9% improvement from baseline to two weeks post-vacation. These findings may suggest that while participants experienced fewer intrusive work-related thoughts, they did not fully disengage mentally from work during their time away. One possible explanation is that detachment may not only be limited to ruminating about work issues but also involves positive work-related thoughts, as proposed by Sonnentag and Fritz (21). For example, detachment and positive connotated problem-solving pondering were found to overlap substantially (27). Following this line of reasoning, some specific “Dailys” activities within the app might have encouraged participants to stay mentally connected to work by reliving positive work experiences or anticipating their return to working life after the vacation. However, since this study did not examine genuinely positive thinking such as positive work reflection (5), this explanation should be regarded as a hypothesis-generating interpretation. Future research could test this mechanism using measures that distinguish constructive work-related thinking (e.g., problem-solving pondering) from maladaptive work-related rumination, alongside established indicators of psychological detachment (88).

### 4.1 Secondary outcomes

The present study focused primarily on work-related rumination as an important transdiagnostic risk factor, along with related mental health outcomes such as depression and insomnia severity. However, for depression, the findings revealed no significant differences between the groups two weeks after the vacation. Although behavioral activation (45), an idea closely related to depression, was included, this approach appeared insufficient to attain significant group differences.

While some studies indicate that targeting transdiagnostic risk factors, such as stress or repetitive negative thinking, show no significant advantage over control conditions in reducing depressive symptoms in the general working population (90), others have reported significant differences (91). This could be partly explained by additional information on depression in the intervention or the inclusion of therapeutic elements such as cognitive restructuring (92). Importantly, meta-analytic evidence suggests that interventions targeting depression through apps typically include longer assessment periods, where medium effects were reported on average at the 11-week follow-up (40). This extended timespan may benefit interventions by allowing more time for the antidepressant effects to unfold. In contrast, the present study only assessed symptom development up to four weeks post-vacation, making it unclear whether the low-intensity Holidaily-app could achieve similar effects on depression. Nevertheless, both groups showed substantial reductions in depressive symptoms following vacation, suggesting that the vacation itself may exert a strong beneficial effect among workers. These findings suggest that, irrespective of the allocated group, taking a vacation can have a meaningful effect on reducing symptoms of depression among workers (93). In a subclinical sample, improvements of 15%–20% may already be considered practically meaningful, whereas in a clinical sample improvements of around 30% are typically regarded as relevant. Against this background, the post-vacation improvements of 21%–45% observed in the present nonclinical sample appear particularly noteworthy (94).

A statistically significant effect on insomnia severity was observed in the intention-to-treat analysis, although this was not replicated in the completer sample. This finding may hint at the relationship between work-related rumination and sleep, which can be explained by cognitive models of insomnia (26, 28, 95). These models emphasize the significant role of heightened negative cognitive activity in the onset and persistence of insomnia symptoms. Importantly, sleep was assessed as a general outcome rather than a specific indicator of pre-sleep cognitive activity. This may have limited the sensitivity of our measurements in detecting changes directly attributable to reduced work-related rumination. Furthermore, intervention effects are commonly noted when participants indicate at least a moderate severity level of insomnia (38). As only 8% of the present sample met the respective threshold, this may have further contributed to the small effect. This is in line with the findings of Miguel et al. (96), where the effects in indicated prevention settings seem to be greater than those in universal ones.

Considering the significance of the DRAMMA framework for intervention design, the absence of intervention effects on DRAMMA-related experiences is noteworthy. One possible explanation could be that the participants may have used the app in a self-directed, competent, and purposeful manner. Although recreational activities, “Dailys,” should be selected or created according to the instructions within Holidaily to foster experiences related to DRAMMA, participants may have used the app primarily to reduce work-related rumination. This could be the result of recruitment measures that emphasize Holidaily as a potential tool to develop the desirable ability to “switch off” mentally from work-related stressors when returning to work after vacation. Thus, self-tailoring, especially digital self-help interventions according to individual needs and preferences, is consistent with the “capable user” concept proposed by Behrendt et al. (97). Furthermore, from a conceptual perspective, experiences related to DRAMMA do not appear to be a prerequisite or mechanism for alleviating work-related rumination; rather, DRAMMA experiences and work-related rumination can be considered separate targets for intervention.

When interpreting the secondary outcomes, it is important to consider the risk of missing real effects (type II error). The study was originally powered for *N* = 788 to identify an effect of *d* = .20, but only 190 participants were included in the analyses. Due to the severely reduced statistical power, it was unlikely that effects of this magnitude, including effects in secondary outcomes, could be detected. It would therefore be premature to conclude that brief, low-threshold interventions cannot have any effect on the secondary outcomes considered in this study.

Overall, these findings may indicate that interventions designed to extend the benefits of vacation primarily influence cognitive recovery processes such as rumination, whereas broader mental health outcomes may require more intensive or longer interventions.

### 4.2 Behavior change

Initially, it was expected that the difference between the intervention and control groups could be explained by the frequency of engaging in recreational activities. This assumption was grounded in previous research showing that frequent engagement in recreational activities extends the positive effects of vacations (7) and reduces repetitive negative thinking (23). However, the present study did not support this hypothesis, as no significant difference was found in the number of recreational activities between the two groups. On average, both groups reported an increase of 2–3 activities between baseline and two weeks post-vacation. Prior studies that aimed to increase the number of recreational activities indicated greater increases in activities and significant group differences in favor of the intervention group (23, 38, 39, 98). This may be due to the scope and content of the questionnaire used to measure recreational activities frequency (57). Although the ReaQ questionnaire seems suitable for assessing recreational activities in daily work life, it may not adequately capture the specific activities that workers commonly engage in before, during, or after returning from vacation when they transition back to their daily work routine. Notably, the Holidaily-app encourages users to participate in activities aimed at extending the positive effects of vacations, such as listening to vacation music, cooking vacation dishes, or reviewing vacation photos. Consequently, the reported quantity of recreational activities by participants may not fully encompass the broader range of activities in which participants actually engaged. To address this limitation, future studies should consider adapting questionnaires to assess vacation-specific recreational activities.

### 4.3 User satisfaction

Regarding satisfaction with the app, 66% of users indicated they would recommend Holidaily to a friend, and 69% reported that they would like to continue using it in their daily working lives. However, these estimates are based on a small subset of the intervention group (32 of 91 participants), which substantially limits the generalizability of the results. Kalon and colleagues (86) reported a higher recommendation rate (77%) for another mental health app, suggesting that stronger satisfaction levels are attainable.

Based on available user experience (UX) data from 36 intervention participants, Holidaily was generally perceived as welcoming, motivating, and pleasant to use, with the highest ratings observed for attractiveness. Overall UX ratings were broadly comparable to those reported for other mobile mental health interventions in studies with similar sample sizes and attrition (99, 100). Nevertheless, across apps, mean UX scores tended to be only slightly to moderately above neutral, indicating room for improvement. This is noteworthy because optimizing UX may represent a relevant lever for strengthening intervention impact, particularly given evidence linking more favorable UX to better mental health outcomes (20).

### 4.4 Limitations and future directions

As the first randomized controlled trial on the effectiveness of a mobile intervention to prolong the positive effects of vacation on employees, the study encountered various challenging obstacles. These issues limit the conclusions of the study but also provide valuable learning experiences for this new field of interventional research.

First, the absence of app-usage data (e.g., login frequency, time spent using the app, or number of activities completed) limits the ability to draw conclusions about the reasons for both intervention and study dropout. Specifically, without usage metrics, it cannot be distinguished whether participants disengaged from the intervention itself or continued using the app but did not complete follow-up surveys. The latter pattern would be consistent with the app being perceived as helpful while questionnaire completion was experienced as overly burdensome, or with participants lacking time and capacity for assessments due to post-vacation return-to-work demands. Evidence from other digital intervention contexts suggests that dropout can reflect a heterogeneous set of motives. For instance, Simon et al. (101) identified an overload of everyday distractions as a primary reason for discontinuation, alongside a wide range of additional explanations. Notably, these included seemingly contradictory accounts, such as perceiving the intervention as not helpful enough vs. discontinuing because personal health goals had already been achieved, an “early completer” pattern described by Christensen et al. (102). Moreover, for digital interventions with relatively simple behavioral goals (e.g., integrating more recreational activities into daily routines), users may quickly internalize the core principles. After a brief initiation phase, participants may feel sufficiently empowered to implement the target behavior independently, reducing the perceived need for continued app-based support. Against this background, Boucher & Raiker (103) argue for a broader conceptualization of engagement that is not restricted to technological usage metrics alone. Specifically, they suggest that engagement should also encompass psychological investment, such as the effort or energy users devote to intervention-relevant tasks prompted by the app content (cf (104).

Second, the final sample size deviated from what was initially intended in the study protocol (50), illustrating a striking “media interest and uptake paradox.” Media attention was extraordinarily high; for example, Germany's most-read health-related magazine (18.71 million readers) published an article on the fading of vacation benefits and included a link to participate. Yet, although the present study is the largest RCT to date on vacations and mental health, participation remained far below expectations. This shortfall substantially reduced statistical power, particularly for secondary outcomes, such that even favorable trends may have gone undetected. This paradox may be partially attributable to an intervention development approach driven by scientists and literature as opposed to a strict user-centered approach. Despite the assumption that workers would welcome a low-threshold tool to mitigate the vacation fade-out effect (11, 14) and the apparent public interest indicated by media coverage, the modest uptake challenges whether the problem is perceived as sufficiently relevant to prompt engagement. It remains uncertain whether workers perceive the fading vacation effect and the associated challenges of recovery, marked by increased work-related rumination, as relevant enough to actively engage in countermeasures. Although this may be part of the explanation, research on the use of digital applications in the context of the COVID-19 pandemic shows that there is a large discrepancy between interest in digital solutions and actual adoption, even for serious, potentially life-threatening health issues. Furthermore, overwhelmed by existing workloads when returning to work, workers may be hesitant to adopt additional interventions, even if they offer potential mental health benefits (101, 105). Although early user experience testing, including feedback from workers and a more rigorous participatory design approach (106), actively seeking feedback on the necessity of an app for addressing the vacation fade-out effect may have provided insightful information. This is an important aspect of future research to help align digital mental health interventions with users' needs and expectations for optimal effectiveness.

Third, substantial attrition, often higher in intervention arms, particularly in larger trials, is a well-documented challenge in mobile mental health research and was also observed in the present study (107, 108). In particular, differential dropout between intervention and control conditions raises concerns about biased estimates. At the same time, it should be considered that dropout-rate differences alone are not a definite indicator of bias since a simulation study by Simon et al. (101) showed that equal dropout does not guarantee unbiased results, and unequal dropout does not necessarily imply bias. The present trial used established methods for handling missing data under Missing Completely at Random (MCAR) and Missing at Random (MAR) assumptions, including multiple imputation and maximum-likelihood mixed models; however, these methods may yield biased estimates if the data are Missing Not at Random (MNAR) (108, 109). Unfortunately, MNAR mechanisms cannot be empirically confirmed from the observed data (108), and the reasons for dropout remain speculative. Assuming a worst-case scenario, bias related to MNAR missingness results from a situation in which participants with high work-related rumination were more likely to discontinue the study. This may lead to an overestimation of the effects if the dropout rate is higher in the intervention group. Although Goldberg et al. (108) found that maximum-likelihood mixed model analyses were robust to MNAR conditions within certain limits, the risk of bias increases as MNAR-driven missingness grows. Therefore, caution is required when interpreting the results. To avoid uncertainty resulting from study dropout, future trials may benefit from reducing the amount of data collected from participants, providing monetary incentives, and incorporating in-person contact with researchers, as meta-analytic evidence suggests that personalized enrollment methods can meaningfully reduce attrition in digital interventions (107).

Fourth, part of the recruitment strategy was to reach many workers through radio and magazine interviews. These interviews provided psychoeducational information concerning improving recovery, reducing work-related rumination, and extending the beneficial vacation effect, which could be accessed by all potential participants in the study. While psychoeducational information has been found to have a relatively weak effect (110), it is still possible to dilute the between-group differences, potentially contributing to the absence of significant group differences in the secondary outcomes.

Finally, while this study's findings indicate that the beneficial vacation effect can be extended, it is important to replicate and validate these findings. Future research should, however, consider that behavior change typically stabilizes between two and three months (111); therefore, outcomes should be assessed at multiple measuring time points (e.g., 6, 8, and 12 weeks post-vacation) to assess participant development.

### 4.5 Conclusion

Overall, this randomized controlled trial provides first evidence that the post-vacation fadeout effect is not inevitable and that at least the positive effects on work-related rumination can be sustained through an intervention. The evidence should be regarded as preliminary due to the limited sample size, substantial attrition, and the relatively follow-up period. Nevertheless, from a universal prevention perspective, and assuming successful scaling, these types of mobile, low-threshold interventions have the potential to promote mental health in the general working population with elevated symptoms of work-related rumination, which is worth exploring further, especially since work-related rumination is an important factor for mental health promotion.

## Data Availability

The datasets presented in this study can be found in online repositories. The names of the repository/repositories and accession number(s) can be found below: https://doi.org/10.48548/pubdata-2096 and https://doi.org/10.48548/pubdata-2101. Further inquiries can be directed to the corresponding author.
